# Research on an Improved Method for Foot-Mounted Inertial/Magnetometer Pedestrian-Positioning Based on the Adaptive Gradient Descent Algorithm

**DOI:** 10.3390/s18124105

**Published:** 2018-11-23

**Authors:** Qiuying Wang, Juan Yin, Aboelmagd Noureldin, Umar Iqbal

**Affiliations:** 1 College of Information and Communication Engineering, Harbin Engineering University, Harbin 150001, China; y18845109628@sina.com; 2 Navigation and Instrumentation Research Group, Electrical and Computer Engineering, Royal Military College of Canada, Kingston, ON K7K 7B4, Canada; noureldin@me.com; 3 Department of Electrical and Computer Engineering, Mississippi State University, Starkville, MS 39759, USA; um_ar@ieee.org

**Keywords:** MIMU, foot-mounted, pedestrian position, fast-initial alignment, heading misalignment angle estimation

## Abstract

Foot-mounted Inertial Pedestrian-Positioning Systems (FIPPSs) based on Micro Inertial Measurement Units (MIMUs), have recently attracted widespread attention with the rapid development of MIMUs. The can be used in challenging environments such as firefighting and the military, even without augmenting with Global Navigation Satellite System (GNSS). Zero Velocity Update (ZUPT) provides a solution for the accumulated positioning errors produced by the low precision and high noise of the MIMU, however, there are some problems using ZUPT for FIPPS, include fast-initial alignment and unobserved heading misalignment angle, which are addressed in this paper. Our first contribution is proposing a fast-initial alignment algorithm for foot-mounted inertial/magnetometer pedestrian positioning based on the Adaptive Gradient Descent Algorithm (AGDA). Considering the characteristics of gravity and Earth’s magnetic field, measured by accelerometers and magnetometers, respectively, when the pedestrian is standing at one place, the AGDA is introduced as the fast-initial alignment. The AGDA is able to estimate the initial attitude and enhance the ability of magnetic disturbance suppression. Our second contribution in this paper is proposing an inertial/magnetometer positioning algorithm based on an adaptive Kalman filter to solve the problem of the unobserved heading misalignment angle. The algorithm utilizes heading misalignment angle as an observation for the Kalman filter and can improve the accuracy of pedestrian position by compensating for magnetic disturbances. In addition, introducing an adaptive parameter in the Kalman filter is able to compensate the varying magnetic disturbance for each ZUPT instant during the walking phase of the pedestrian. The performance of the proposed method is examined by conducting pedestrian test trajectory using MTi-G710 manufacture by XSENS. The experimental results verify the effectiveness and applicability of the proposed method.

## 1. Introduction

Pedestrian Position Systems based on Micro Inertial Measurement Unit (MIMUs) have recently attracted widespread attention with the rapid development of MIMUs. Foot-mounted MIMUs (including gyroscopes and accelerometers) can measure the acceleration and angular velocity of pedestrians in real time. These measurements are utilized to estimate velocity and position. MIMUs are self-contained fully autonomous systems, immune to external interference. They can be used in Global Navigation Satellite System (GNSS)-denied environments, and are very beneficial in firefighting and military applications [1,2]. However, the positioning accuracy of MIMUs deteriorates because of sensors’ bias, drift, misalignment, scale factors, instability, and high noise during the dead reckoning process [3,4].

Installing the MIMU on the pedestrian foot is called Foot-mounted Inertial Pedestrian-Positioning System (FIPPS) [5,6,7,8]. A Kalman filter (KF) is utilized to estimate each navigation error to reduce the positioning error caused by measurement noise of inertial components. Theoretically, the velocity of the foot is zero when the pedestrian’s foot touches the ground momentarily while walking. In the absence of measurement noise during the stationary state of the pedestrian foot while walking ideally both the acceleration and the calculated velocity should be zeroed. However, due to the measurement noise of the sensors, the velocity is not zero. To enhance the performance of KF by compensating the velocity error during the stationary state of the pedestrian foot an innovative algorithm called Zero Velocity Update (ZUPT) was introduced [9,10,11,12,13,14,15]. Nonetheless, there are some problems using ZUPT for FIPPS to correct the accumulated positioning errors.

The first problem for FIPPS is the initial value setting of the dead reckoning algorithm. The principle of dead reckoning is that the increment (including position increment, velocity increment and attitude increment) is obtained by the MIMU measurement. Then, the navigation information could be calculated by the sum of the initial value and the increment. It is assumed that the pedestrian’s initial state is standing at one point, so the initial velocity value is zero. The initial position can be obtained by GNSS or set to zero. The difficulty for FIPPS is the determining the initial attitude. The process of obtaining the initial attitude is called initial alignment. To acquire the initial alignment for a high precision Inertial Navigation System (INS), it will be assumed that an accelerometer will measure only gravity, a gyro will measure only the Earth’s rotation angle velocity [16,17]. However, for low cost MIMU the measurement noise of gyro is much larger than the Earth’s rotation angle velocity, therefore, the initial alignment algorithms for high precise INS are not suitable for FIPPS. The Rotation Modulation Technique is an option for initial alignment of MIMUs but a rotating platform is needed, and the initial alignment time is too lengthy [18,19,20,21,22]. Nilsson et al. [23] proposed to a MIMU initial alignment algorithm based on accelerometers and magnetometers. Nevertheless, local magnetic distortions may still corrupt the estimated heading, and the alignment accuracy can be further decreased by magnetic interference.

The second problem for FIPPS is the observability of ZUPT. ZUPT can reduce the position error, velocity error, and horizontal misalignment angle of the system, but the heading misalignment angle is unobserved, which results in a continuous increase in the heading error and an increase in the positioning error. The heading errors can be corrected by augmenting MIMU with other sensors [24,25]. Magnetometers are some of the most commonly used sensors. A magnetometer is a sensor that measures the intensity of a magnetic field in the local coordinate system. In theory, the heading can be obtained using the magnetic field strength measurement. Conversely, due to the presence of magnetic interference, the heading conversion is inaccurate, and it cannot be used as a reference to locate the misalignment angle [26,27]. Intrinsic calibration is one of the magnetometer calibration processes. The most popular magnetometer intrinsic calibration approach is attitude-independent method, which exploits the fact that the magnetometer measurement at the local position or in a homogeneous magnetic field is constant in magnitude regardless of the orientation [28,29,30,31,32,33,34,35,36]. 

To address the limitation of correction schemes using the magnetometer discussed above, an improved method for foot-mounted inertial/ magnetometer pedestrian positioning based on adaptive gradient descent algorithm is proposed in this paper. This paper consists of the following sections: Section 2 introduces the principle of the foot-mounted inertial pedestrian-positioning system. Observability during the ZUPT period is carried out to lay a foundation of further study on heading misalignment angle estimation. Section 3 introduces the fast-initial alignment based on an adaptive gradient descent algorithm, and the measurement interference is estimated at the end of initial alignment. This is the first contribution in this paper. Section 4 introduces an adaptive inertial/magnetometer positioning algorithm by improving heading observability that is able to address the limitation of estimating the heading misalignment angle by the traditional ZUPT method. This is the second contribution. A scheme of the improved pedestrian position algorithm is shown in Section 5. Performance of the proposed method is tested using MTi-710 in the same section. The last part draws the conclusions.

## 2. Principle of Foot-Mounted Inertial Pedestrian-Positioning System 

### 2.1. Dead Reckoning for Inertial Pedestrian Positioning

For FIPPS, the MIMU is installed on the pedestrian foot, and the acceleration and angular velocity of the pedestrian foot are measured in real time. Then, the pedestrian positioning information is obtained through the dead reckoning method [37]. The core formula of dead reckoning is as follows:

(1) $$\{\begin{array}{l} C_{b}^{n}{}_{k}=C_{b}^{n}{}_{\left(k-1\right)}\left(I+\Omega_{k}T\right)\\ v_{k}^{n}=v_{k-1}^{n}+\left(C_{b}^{n}{}_{k}f_{k}^{b}+g^{n}\right)T\\ p_{k}^{n}=p_{k-1}^{n}+v_{k}^{n}T+\left(C_{b}^{n}{}_{k}f_{k}^{b}+g^{n}\right)T^{2}/2 \end{array}$$

Here, subscripts k (k=1,⋯,N) are the sample time; $C_{b}^{n}{}_{k}$ is the transformation matrix between the sensor frame (short for b frame, right-front-up frame) and the navigation frame (short for n frame, east-north-up frame) at time *k*; I is unit matrix; T is sample time; $\Omega_{k}=\left[\omega_{k}\times \right]$ is antisymmetric matrix of angular velocity $\omega_{k}$ measured by gyro; $v_{k}^{n}$ is the velocity along n frame at time *k*; $f_{k}^{b}$ is acceleration measured by the accelerometer along b frame; $g^{n}={\left[\begin{array}{ccc} 0 & 0 & -g \end{array}\right]}^{T}$ is a projection of gravity along the *n* frame; $p_{k}^{n}$ is calculating position result along *n* frame.

**Figure 1:**
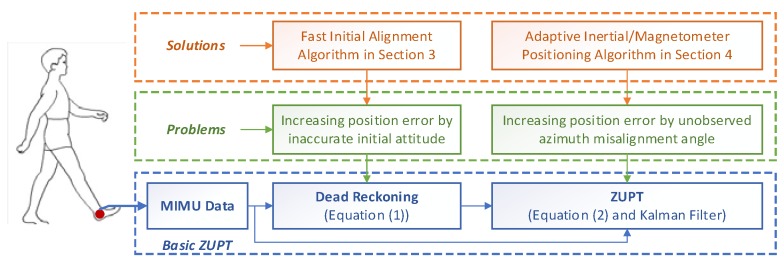
The relationships between basic ZUPT, its problems and the proposed algorithms.

From Equation (1), it can be obtained that setting the parameters of $v_{0}^{n}=O_{3\times 1}$, $p_{0}^{n}=O_{3\times 1}$ and $C_{b}^{n}{}_{0}$ is the first step of FIPPS. Hence, if $C_{b}^{n}{}_{0}$, which is set by initial alignment, is inaccurate, the position error will be increased. For high precise INS initial alignment, it is assumed that only gravity and the Earth’s rotation angle velocity can be measured by accelerometers and gyros, respectively. However, for a low cost MIMU, the measurement noise of gyro is much larger than the Earth’s rotation angle velocity. Therefore, the initial alignment algorithm for a high precision INS is not suitable for FIPPS. To solve the problem, a MIMU initial alignment algorithm based on accelerometers and magnetometers is proposed. In practice, local magnetic distortions may still corrupt the estimated heading, and the alignment accuracy is further decreased by magnetic interference. Hence, how to set the initial value of $C_{b}^{n}{}_{0}$ with the magnetic interference (shown in Figure 1) will be discussed in Section 3.

### 2.2. Basic ZUPT Algorithm

The low-cost MEMS-based MIMU sensors have high noise and composite error characteristics. The overall positioning accuracy deteriorates due to the measurement error of the $\Omega_{k}$ and $f_{k}^{b}$ in Equation (1). The positional errors are accumulated and enlarged with the passage of time, which significantly reduces the pedestrian tracking results.

The ZUPT scheme is utilized to address this problem [38,39,40]. Considering the motion features of the pedestrian foot, it is assumed that the foot is in a stationary state when the foot is touching the ground. Using the error equation of Equation (1) as the state equation during the period of foot is in-touch with ground, and using velocity error as an observation. The system state equation and measurement equation are established:

(2) $$\begin{array}{l} \dot{X}=AX+\eta_{k}\\ Z=HX+\nu_{k} \end{array}$$

where, $X={\left[\begin{array}{ccccc} \delta p^{T} & \delta v^{T} & \Phi^{T} & \Delta^{T} & \varepsilon^{T} \end{array}\right]}^{T}$ is the state of the system, $\delta p={\left[\begin{array}{ccc} \delta p_{x} & \delta p_{y} & \delta p_{z} \end{array}\right]}^{T}$, $\delta v={\left[\begin{array}{ccc} \delta v_{x} & \delta v_{y} & \delta v_{z} \end{array}\right]}^{T}$, $\Phi ={\left[\begin{array}{ccc} \phi_{x} & \phi_{y} & \phi_{z} \end{array}\right]}^{T}$ are the position error, velocity error, and misalignment angle respectively; Δ, ε are the accelerometer bias and gyro drift, respectively; $H=\left[\begin{array}{lllll} O_{3\times 3} & I_{3\times 3} & O_{3\times 3} & O_{3\times 3} & O_{3\times 3} \end{array}\right]$ is the measure matrix; Z is the observation; $\eta_{k}$, $\nu_{k}$ are state noise and measurement noise respectively; A is the system transfer matrix and the specific form is as follows:

$$A=\left[\begin{array}{ccccc} O_{3\times 3} & I_{3\times 3} & O_{3\times 3} & O_{3\times 3} & O_{3\times 3}\\ O_{3\times 3} & O_{3\times 3} & \left[f^{n}\times \right] & C_{b}^{n} & O_{3\times 3}\\ O_{3\times 3} & O_{3\times 3} & O_{3\times 3} & O_{3\times 3} & -C_{b}^{n}\\ O_{3\times 3} & O_{3\times 3} & O_{3\times 3} & O_{3\times 3} & O_{3\times 3}\\ O_{3\times 3} & O_{3\times 3} & O_{3\times 3} & O_{3\times 3} & O_{3\times 3} \end{array}\right]$$

where $\left[f^{n}\times \right]$ represents antisymmetric matrix of $f^{n}$, and $f^{n}=C_{b}^{n}f^{b}$.

Then, based on this observation, the Kalman filter is used to estimate each navigation error to reduce the positioning error caused by measurement noise of inertial components [41,42]. The algorithm according to Equations (1) and (2) is called basic ZUPT, which is the basic algorithm and used as the compare algorithm in the experiment section.

It can be found that matrix A will not change during the period of foot is in-touch with ground. Therefore, Equation (2) is a linear time-invariant system. There is an observability analysis theorem for linear time-invariant system.

**Theorem** **1.** *If the following condition can be satisfied, a linear time-invariant system is a completely observable system:*

$$rank\left[\begin{array}{cccc} H^{T} & A^{T}H^{T} & \cdots & {\left(A^{T}\right)}^{j-1}H^{T} \end{array}\right]=j$$

*where*, j*is the dimension.*

According to the theorem, it can be calculated that the rank of the matrix is 5 and five variables of X can be estimated. Essentially the velocity error can be estimated because the observation is velocity error. Hence, other two variables of δp and Φ are observed. According to references [43,44], the horizontal misalignment angles can be estimated by ZUPT. Therefore, part of the position error caused by velocity error and the horizontal misalignment angles can be compensated. However, the part of the position error caused by the heading misalignment angle cannot be corrected. It means that the position error caused by the heading cannot be compensated by the basic ZUPT algorithm. The solution for the heading misalignment angle problem will be discussed in Section 4. Figure 1 shows the relationship between the basic ZUPT algorithm, the problems of ZUPT and the proposed algorithms in this paper.

## 3. Fast-Initial Alignment Based on Adaptive Gradient Descent Algorithm

The purpose of initial alignment for pedestrian alignment is to obtain the initial attitude within a few seconds. It is hard to finish the initial alignment only using MIMUs themselves because of their high measurement noise. Hence, introducing the magnetometer as an external sensor to the initial alignment is proposed in this paper. 

Madgwick [45] proposed a novel orientation filter that can obtain the attitude information by Gradient Descent Algorithm (GDA). The filter uses a quaternion representation, allowing accelerometer and magnetometer data to be used in an analytically derived and optimized gradient-descent algorithm to compute the direction of the gyroscope measurement error as a quaternion derivative. Therefore, GDA can be introduced as the initial alignment algorithm for FIPPS.

However, the precondition of introducing GDA for initial alignment is that the accelerometer and magnetometer errors are neglected [45]. The measurement error of accelerometers comes from the sensor itself and can be compensated beforehand, but local magnetic distortions may still corrupt the estimated heading. Hence, the attitude estimation accuracy of GDA will be influenced by local magnetic distortions. 

**Figure 2:**
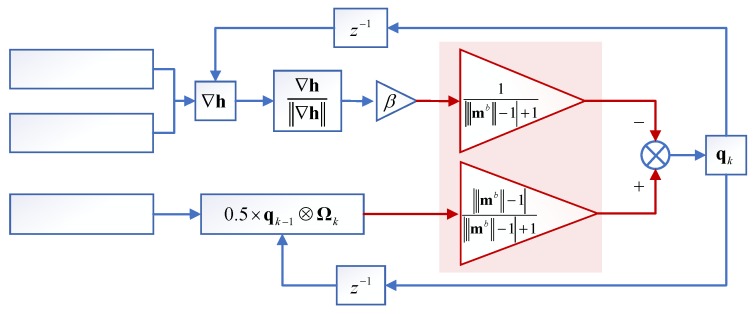
Diagram of AGDA for pedestrian initial alignment.

The Adaptive Gradient Descent Algorithm (AGDA), which is used to enhance the ability of magnetic disturbance suppression, is proposed to the fast-initial alignment for pedestrian position system. The diagram of AGDA is shown in Figure 2. The core formula of AGDA is as follows:

(3a) $$\nabla h=\left[\begin{array}{c} J_{g}^{T}h_{g}\\ J_{g,b}^{T}h_{g,b} \end{array}\right]$$

(3b) $${\dot{q}}_{\Delta k}=\frac{\nabla h}{\left\|\nabla h\right\|}$$

(3c) $${\dot{q}}_{\omega k}=\frac{1}{2}\times q_{k-1}⊗\Omega_{k}$$

(3d) $${\dot{q}}_{k}=\frac{\left|\left\|m^{b}\right\|-1\right|}{\left|\left\|m^{b}\right\|-1\right|+1}\cdot {\dot{q}}_{\omega k}-\frac{1}{\left|\left\|m^{b}\right\|-1\right|+1}\cdot \beta {\dot{q}}_{\Delta k}$$

(3e) $$q_{k}=q_{k-1}+{\dot{q}}_{k}T$$

Here, $q_{k}$, $q_{k-1}$ are quaternion at time *k*, *k* − 1 respectively. ${\dot{q}}_{k}$ is differential of $q_{k}$ at time k. ${\dot{q}}_{\omega k}$ is differential of $q_{\omega k}$ calculated by the angular velocity measured by gyro, the calculating formula is shown in Equation (3c). β is a weight parameter. $m^{b}={\left[\begin{array}{ccc} m_{x}^{b} & m_{y}^{b} & m_{z}^{b} \end{array}\right]}^{T}$ is the magnetometer measurement with normalized along the b frame. $\left\|m^{b}\right\|=\sqrt{m_{x}^{b2}+m_{y}^{b2}+m_{z}^{b2}}$, and $m_{i}^{b}$ (i=x,y,z) is the magnetometers measurement with normalized along the b frame. ${\dot{q}}_{\Delta k}$ is the differential of $q_{\Delta k}$ calculated by AGDA, the calculating formula is shown in Equation (6), the form of ∇h is shown in Equation (4). The forms of $h_{g}$, $h_{g,b}$, $J_{g}^{}$, $J_{g,b}^{}$ are as follows:

$$J_{g,b}^{}=\left[\begin{array}{c} J_{g}^{T}\\ J_{b}^{T} \end{array}\right],\;J_{g}^{}=[\begin{array}{cccc} -2q_{3} & 2q_{4} & -2q_{1} & 2q_{2}\\ 2q_{2} & 2q_{1} & 2q_{4} & 2q_{3}\\ 0 & -4q_{2} & -4q_{3} & 0 \end{array}],$$

$$J_{b}^{}=[\begin{array}{cccc} -2m_{z}^{n}q_{3} & 2m_{z}^{n}q_{4} & -4m_{x}^{n}q_{3}-2m_{z}^{n}q_{1} & -4m_{x}^{n}q_{3}+2m_{z}^{n}q_{2}\\ -2m_{x}^{n}q_{4}+2m_{z}^{n}q_{2} & 2m_{x}^{n}q_{3}+2m_{z}^{n}q_{1} & 2m_{x}^{n}q_{2}+2m_{z}^{n}q_{4} & -2m_{x}^{n}q_{1}+2m_{z}^{n}q_{3}\\ 2m_{x}^{n}q_{3} & 2m_{x}^{n}q_{4}-4m_{z}^{n}q_{2} & 2m_{x}^{n}q_{1}-4m_{z}^{n}q_{3} & 2m_{x}^{n}q_{2} \end{array}],$$

$$h_{g,b}=\left[\begin{array}{c} h_{g}\\ h_{b} \end{array}\right],\;h_{g}=[\begin{array}{c} 2\left(q_{2}q_{4}-q_{1}q_{2}\right)-f_{x}^{b}\\ 2\left(q_{1}q_{2}+q_{3}q_{4}\right)-f_{y}^{b}\\ 2\left(0.5-q_{2}^{2}-q_{3}^{2}\right)-f_{z}^{b} \end{array}],$$

$$h_{b}=\left[\begin{array}{c} 2m_{x}^{n}\left(0.5-q_{3}^{2}-q_{4}^{2}\right)+2m_{z}^{n}\left(q_{2}q_{4}-q_{1}q_{2}\right)-m_{x}^{b}\\ 2m_{x}^{n}\left(q_{2}q_{3}-q_{1}q_{4}\right)+2m_{z}^{n}\left(q_{1}q_{2}+q_{3}q_{4}\right)-m_{y}^{b}\\ 2m_{x}^{n}\left(q_{1}q_{3}+q_{2}q_{4}\right)+2m_{z}^{n}\left(0.5-q_{2}^{2}-q_{3}^{2}\right)-m_{z}^{b} \end{array}\right]$$

where, $f^{b}={\left[\begin{array}{ccc} f_{x}^{b} & f_{y}^{b} & f_{z}^{b} \end{array}\right]}^{T}$ is the accelerometer measurement. $m^{n}={\left[\begin{array}{ccc} m_{x}^{n} & m_{y}^{n} & m_{z}^{n} \end{array}\right]}^{T}$ is the Earth’s magnetic field along *n* frame, $m_{x}^{n}=\cos\kappa$, $m_{y}^{n}=0$, $m_{z}^{n}=\sin\kappa$, κ the magnetic inclination related with latitude. It can be found in the literature.

According to the Figure 2 and Equation (3d), the adaptive adjusting parameters of $\left|\left\|m^{b}\right\|-1\right|/\left(\left|\left\|m^{b}\right\|-1\right|+1\right)$ and $1/\left(\left|\left\|m^{b}\right\|-1\right|+1\right)$ are introduced to reduce the effect of magnetic disturbance on the initial alignment, which is the difference with the GDA by Madgwick [45]. If there is no magnetic disturbance, $\left\|m^{b}\right\|=1$ and the adaptive adjusting parameters could not be activated. Hence, ${\dot{q}}_{k}=-\beta {\dot{q}}_{\Delta k}$. It means that ${\dot{q}}_{k}$ is only updated by ${\dot{q}}_{\Delta k}$. The value of $\left|\left\|m^{b}\right\|-1\right|$ and $\left|\left\|m^{b}\right\|-1\right|/\left(\left|\left\|m^{b}\right\|-1\right|+1\right)$ will increase with the increase of magnetic disturbance, while the value of $1/\left(\left|\left\|m^{b}\right\|-1\right|+1\right)$ decreases. Therefore, the weight of ${\dot{q}}_{\Delta k}$ for updating the ${\dot{q}}_{k}$ decreases, and the weight of ${\dot{q}}_{\omega k}$ increases. The effects of magnetic disturbance on initial alignment can be reduced.

The optimal solution of the quaternion is obtained by Equation (3). The transformation matrix of $C_{b0}^{n}$ is determined using the relationship between the quaternion and the transformation matrix presented in Appendix A.

After initial alignment, the magnetic disturbance at the pedestrian standing point is calculated based on the initial alignment result $C_{b0}^{n}$ and the local magnetic $m^{n}$ as follows:

(4) $$\delta m^{n}=m^{n}-C_{b}^{n}{}_{0}m^{b}$$

where, $\delta m^{n}={\left[\begin{array}{ccc} \delta m_{x}^{n} & \delta m_{y}^{n} & \delta m_{z}^{n} \end{array}\right]}^{T}$ is the estimation magnetic disturbance along the n frame by AGDA.

Two key advantages of introducing AGDA as the fast-initial alignment algorithm are: (1) the magnetic disturbance can be estimated and compensated in a short time; (2) the initial attitude can be obtained by the accelerometer and magnetometer measurement without magnetic disturbance. Therefore, the AGDA can be used as the initial alignment algorithm, but it cannot be used in the ZUPT during the pedestrian walking period. Because the duration of time foot touches the ground while walking is about 1 s or shorter.

To calculate the uncertainty of the AGDA, the difference between the earth’s magnetic field and the magnetometer measurement after compensation can be introduced as the criteria. Equation (5) is the calculation formula of the AGDA uncertainty:

(5) $$\tau =\left\|m^{n}\right\|-\left\|m^{b}-C_{n}^{b}\delta m^{n}\right\|$$

where, τ is the uncertainty value; ‖•‖ is the module value of the vector; $C_{n}^{b}$ is the initial alignment result in real time.

For the AGDA, it is assumed that the Earth’s magnetic field at the same place is constant. Hence, if both initial alignment result $C_{n}^{b}$ and the magnetic distribution estimation result are accurate, τ is zero. The value of τ will increase when the initial alignment result and the magnetic distribution estimation results are inaccurate. Then, τ can be used to calculate the uncertainty in AGDA.

## 4. Adaptive Inertial/Magnetometer Positioning Algorithm by Improving Heading Observability

According to the principle of magnetometers, the heading can be obtained by the magnetometer measurement when the magnetic interference is corrected. Then, for the Inertial/Magnetometer Positioning system, the heading information can be calculated in real time when the magnetic interference is estimated during the initial alignment process. However, the magnetic interference can be inconsistent at different points of the trajectory. For example, magnetic fields produced by a power line along the walking trajectory will change the magnetic disturbance. Hence, the magnetic disturbance at ZUPT point during the pedestrian walking trajectory is different from that at an initial alignment point. This section discusses the important issue of how to estimate and reduce the magnetic disturbance.

### 4.1. Magnetic Interference Compensated and Heading Estimation

Based on the observation analysis in Section 2.2, the horizontal misalignment angles can be estimated by Kalman Filter for ZUPT. Hence, the magnetic measured by the magnetometers can be transformed to the horizontal frame (Equation (6)) based on the relationship between the Euler angles and the transformation matrix (Appendix A):

(6) $$m^{\tilde{n}}=C_{y}C_{x}m^{b}$$

Here, the difference between the $\tilde{n}$ and b frame is two horizontal attitude rotations: the first rotation is about the x-axis through an angle ${\tilde{\varphi }}_{x}$, the second is about the y-axis through an angle ${\tilde{\varphi }}_{y}$ from b frame to $\tilde{n}$ frame. The difference between the n frame and the $\tilde{n}$ frame is the heading ${\tilde{\varphi }}_{z}$. The matrix of $C_{x}$ and $C_{y}$ are composed of ${\tilde{\varphi }}_{x}$ and ${\tilde{\varphi }}_{y}$, respectively as shown in Appendix A. $m^{\tilde{n}}={\left[\begin{array}{ccc} m_{x}^{\tilde{n}} & m_{y}^{\tilde{n}} & m_{z}^{\tilde{n}} \end{array}\right]}^{T}$ is the magnetic field along the $\tilde{n}$ frame after transformation.

**Figure 3:**
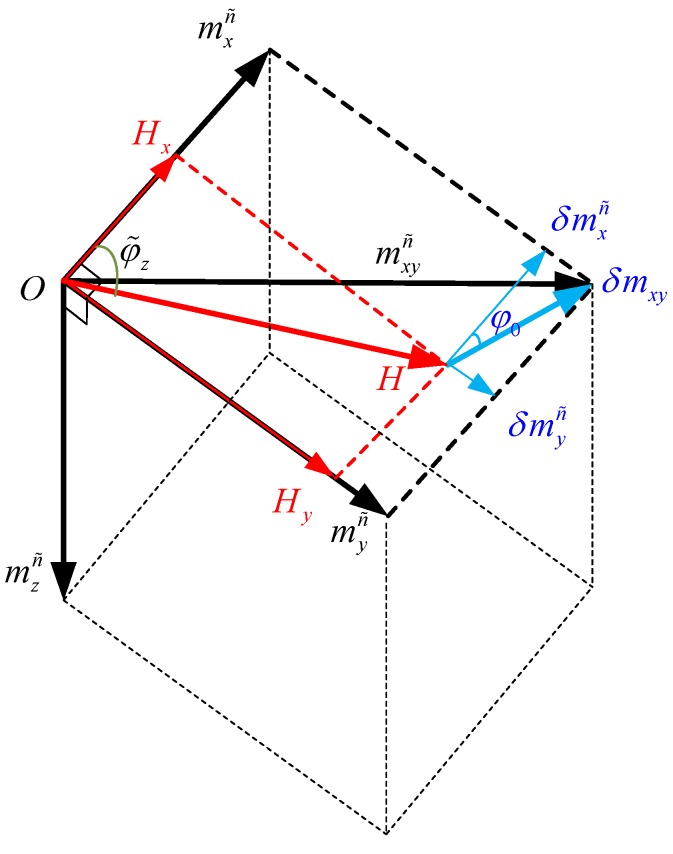
The geometric relation of earth magnetic field, magnetic disturbance and magnetometer magnetic (magnitude and direction of the magnetic field).

The geometric relation of Earth’s magnetic field, magnetic disturbance, and magnetometer magnetic (magnitude and direction of the total magnetic field) measured by the magnetometer is shown in Figure 3. Because the heading information can be obtained by the magnetic projection in the horizontal plane, only relation in the plane is shown. The main purpose of this section is the determination of heading ${\tilde{\varphi }}_{z}$.

Here, H=cosκ is the projection of the local Earth magnetic field along the magnetic north, and it can be obtained by lookup. $\delta m_{xy}^{n}=\sqrt{\delta m_{x}^{n}{}^{2}+\delta m_{y}^{n}{}^{2}}$ and $\delta m_{x}^{n}$, $\delta m_{y}^{n}$ are calculated by Equation (4).

According to the geometric relation in Figure 3, the mathematic relationship between the Earth’s magnetic field, magnetic disturbance and magnetometer magnetic along the $\tilde{n}$ frame is as follows,

(7) $$\{\begin{array}{l} m_{x}^{\tilde{n}}=H_{x}+\delta m_{x}^{\tilde{n}}=H\cos{\tilde{\varphi }}_{z}+\delta m_{xy}\cos\varphi_{0}\\ m_{y}^{\tilde{n}}=H_{y}+\delta m_{y}^{\tilde{n}}=H\sin{\tilde{\varphi }}_{z}+\delta m_{xy}\sin\varphi_{0} \end{array}$$

where, ${\tilde{\varphi }}_{z}$ is the heading between $m_{x}^{\tilde{n}}$ and H. $\varphi_{0}$ is the angle between $\delta m_{xy}$ and $m_{x}^{\tilde{n}}$.

The heading ${\tilde{\varphi }}_{z}$ and the angle $\varphi_{0}$ can be obtained by solving Equation (7). Appendix B shows the derivation process.

(8) $$\{\begin{array}{l} {\tilde{\varphi }}_{z}=\arcsin\frac{m_{x}^{\tilde{n}}{}^{2}+m_{y}^{\tilde{n}}{}^{2}+H^{2}-\delta m_{xy}^{2}}{2H\sqrt{m_{x}^{\tilde{n}}{}^{2}+m_{y}^{\tilde{n}}{}^{2}}}-\arcsin\frac{m_{x}^{\tilde{n}}}{\sqrt{m_{x}^{\tilde{n}}{}^{2}+m_{y}^{\tilde{n}}{}^{2}}}\\ \varphi_{0}=\arcsin\frac{m_{x}^{\tilde{n}}{}^{2}+m_{y}^{\tilde{n}}{}^{2}+\delta m_{xy}^{2}-H^{2}}{2\delta m_{xy}^{}\sqrt{m_{x}^{\tilde{n}}{}^{2}+m_{y}^{\tilde{n}}{}^{2}}}-\arcsin\frac{m_{x}^{\tilde{n}}}{\sqrt{m_{x}^{\tilde{n}}{}^{2}+m_{y}^{\tilde{n}}{}^{2}}} \end{array}$$

${\tilde{\varphi }}_{z}$ in Equation (8) is the heading result by the magnetometer measurement after the magnetic disturbance is corrected during pedestrian ZUPT segments of the trajectory. However, it cannot be used as the heading information because measurement noise is included. The solution is discussed in Section 4.2.

### 4.2. Inertial/Magnetometer Positioning Algorithm Based on Adaptive Kalman Filter

The heading misalignment angles $\phi_{z}$ can be obtained by the difference between the magnetometer heading (Equation (8)) and gyro heading (Equation (1)). Then, $\phi_{z}$ can be introduced as the new observation for the Kalman filter of ZUPT, which is used to estimate heading misalignment angle and solving the unobserved variable’s $\phi_{z}$ problem. This results in a reduction of the position error caused by heading errors.

However, the premise of solution above is that the magnetic disturbance $\delta m^{n}$ obtained during the initial alignment is suitable for ZUPT during pedestrian walking. It means that the magnetic disturbance at two stand points (initial alignment point and ZUPT point during walking) will remain the same. However, the magnetic disturbance is variable at any time. For example, the magnetic disturbance will be changed by a power line along the walk way of the trajectory. Therefore, introducing an adaptive parameter in the Kalman filter is a solution to the problem above. The core formula of adaptive Kalman filter is as follows:

(9a) $${\hat{X}}_{k}^{-}=\Phi_{k}{\hat{X}}_{k-1}$$

(9b) $$P_{k}^{-}=\Phi_{k}P_{k-1}\Phi_{k}^{T}+Q_{k}$$

(9c) $$K_{k}=P_{k}^{-}H_{k}^{T}{\left(H_{k}P_{k}H_{k}^{T}+\alpha R_{k}\right)}^{-1}$$

(9d) $${\hat{X}}_{k}={\hat{X}}_{k}^{-}+K_{k}(Z_{k}-H_{k}{\hat{X}}_{k}^{-})$$

(9e) $$P_{k}=(I-K_{k}H_{k})P_{k}^{-}$$

Here, Φ is the discretization matrix of A in Equation (1). α is the adaptive parameter. H form is:

$$H=\left[\begin{array}{lllll} O_{3\times 3} & I_{3\times 3} & O_{3\times 3} & O_{3\times 3} & O_{3\times 3}\\ O_{1\times 3} & O_{1\times 3} & E_{1\times 3} & O_{1\times 3} & O_{1\times 3} \end{array}\right]$$

Here, $E_{1\times 3}=\left[\begin{array}{ccc} 0 & 0 & 1 \end{array}\right]$. 

The adaptive parameter α in Equation (9c) is calculated by the following formula:

(10a) $$E_{k}=\left(Z_{k}-H_{k}{\hat{X}}_{k}^{}\right){\left(Z_{k}-H_{k}{\hat{X}}_{k}^{}\right)}^{T}$$

(10b) $${\bar{E}}_{k}=\frac{1}{M-1}\sum_{j=k-M+1}^{M}\left(Z_{j}-H_{k}{\hat{X}}_{k}^{}\right){\left(Z_{j}-H_{k}{\hat{X}}_{k}^{}\right)}^{T}$$

(10c) $$\alpha =\left|tr\left(E_{k}\right)-tr({\bar{E}}_{k})\right|$$

where, M is the length of the average, and its value is related with the sample time.

**Figure 4:**
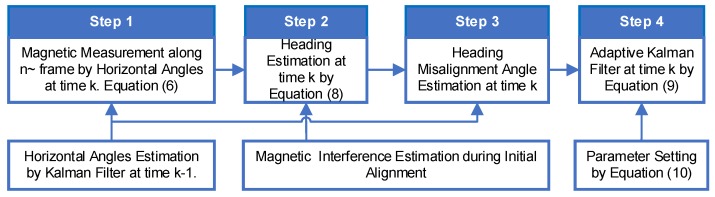
Adaptive Inertial/Magnetometer Positioning Algorithm.

When the magnetic disturbance at ZUPT point is different from the initial alignment point, the magnetic projection measured by the magnetometers along the $\tilde{n}$ frame $m^{\tilde{n}}={\left[\begin{array}{ccc} m_{x}^{\tilde{n}} & m_{y}^{\tilde{n}} & m_{z}^{\tilde{n}} \end{array}\right]}^{T}$ will change. If $E_{k}$ increases, then, the difference between the parameter ($E_{k}$) and average ${\bar{E}}_{k}$ will be increased by α. The observation noise matrix R should be increased. Therefore, the multiplication of α and R is introduced into the Kalman filter to reduce the effect of magnetic disturbance change (Equation (9c)). Figure 4 is the block diagram of the adaptive Inertial/Magnetometer positioning algorithm proposed in this section. According to the principle of Kalman Filter, the $P_{k}$ can be used to judge the uncertainty of the adaptive Inertial/Magnetometer positioning algorithm.

## 5. Scheme of Improved Pedestrian Position Algorithm and Performance Evaluation

### 5.1. Scheme of Improved Pedestrian Position Algorithm

**Figure 5:**
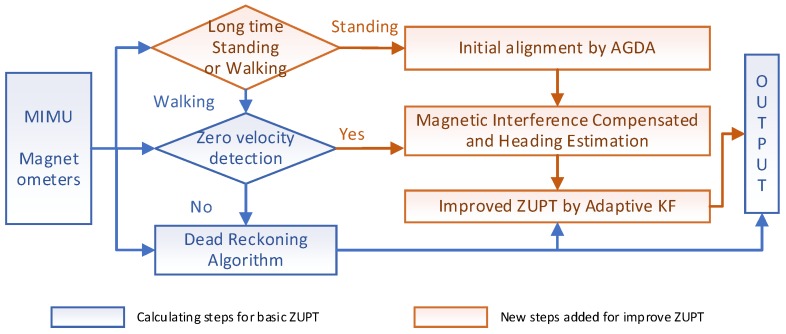
Schematic diagram of the improved algorithm for FIPPS.

According to the traditional pedestrian positioning method mentioned in Section 2, Figure 5 shows a schematic diagram of the improved pedestrian positioning algorithm, including the fast-initial alignment algorithm and an adaptive inertial/Magnetometer ZUPT positioning algorithm mentioned in Section 3 and Section 4.

The blue boxes in Figure 5 are the characteristic calculation steps for a basic ZUPT. The orange boxes are the proposed new steps to improve the ZUPT algorithm in this paper. Firstly, initial attitude and the magnetometer interference are estimated by AGDA, which is introduced as the initial alignment algorithm for FIPPS. AGDA can only be used as the initial alignment algorithm, not for ZUPT. Because the ZUPT time is too short, the parameters cannot be estimated by ADGA. Secondly, based on the difference between the magnetometer heading and gyro heading, the heading misalignment angle is introduced as the new observation for the Kalman filter during ZUPT period. In addition, the problem of the change of the magnetic disturbance by surrounding environment is solved by introducing an adaptive parameter in the Kalman filter. These innovative changes enhance position accuracy as the heading error is reduced consistently.

### 5.2. Experiment Study

To verify the correctness and effectiveness of the improved method for foot-mounted inertial/ magnetometer pedestrian-positioning system based on AGDA in this paper, MTi-G710 MIMU manufactured by XSENS (Enschede, Netherlands) is used as the footwear test equipment for walking experiments. Table 1 shows the parameters of the MTi-G710. 

**Figure 6:**
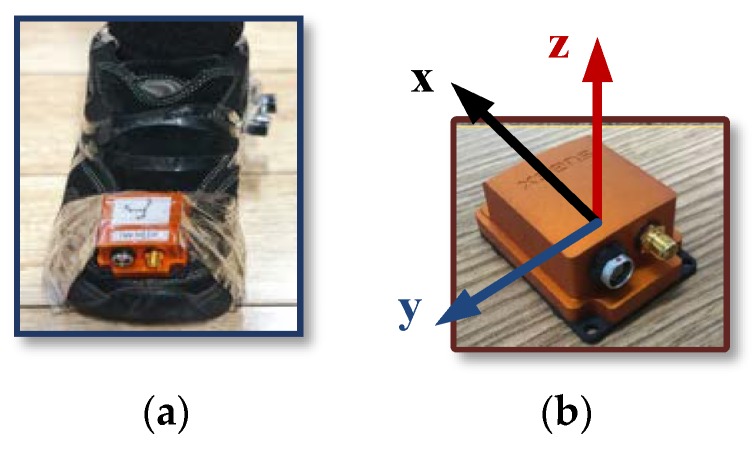
The position and the orientation of externally located MTI-710 on Shoe. (**a**) MTi-G710 installed on the foot; (**b**) MTi-G710 and its frame.

The position and the orientation of the MIMU connected to a shoe are shown in Figure 6 to collect the output of the accelerometers, gyroscopes, magnetometers and GPS. The data collected by accelerometers, gyroscopes and magnetometers is utilized to calculate the positioning results of the pedestrian in real time. GPS position is the standard information. The sample frequency is 100 Hz.

Two test trajectories were carried out. Trajectory 1 was conducted to test and verify the validity of the algorithm proposed in this paper. For the first trajectory, an “8 shape” path was chosen, and there was no change in the magnetic interference throughout this trajectory. The second trajectory was conducted around the buildings to test the stability of the improve ZUPT algorithm, especially in the changing magnetic interference scenarios. Building proximity introduced many variable factors to influence the magnetometer interference, which included electric cables, metals, etc.

Test 1

**Figure 7:**
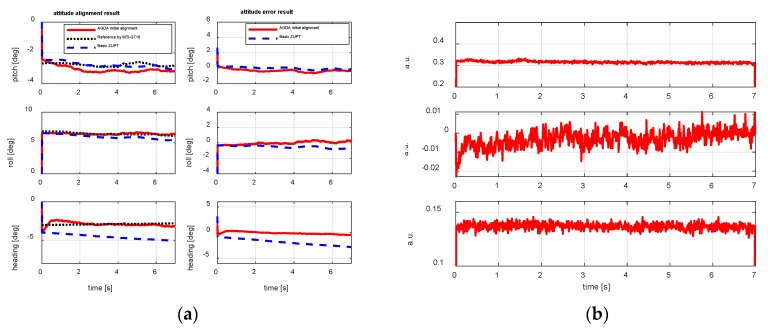
Attitude and magnetic disturbance estimation by initial alignment. (**a**) Attitude result; (**b**) Magnetic disturbance.

**Figure 8:**
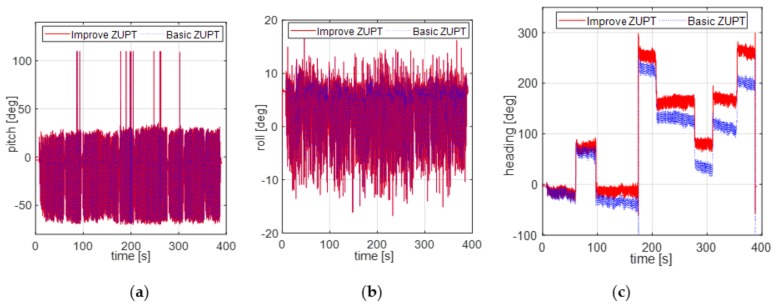
Pedestrian heading estimation result. (**a**) Pitch; (**b**) Roll; (**c**) Heading.

**Figure 9:**
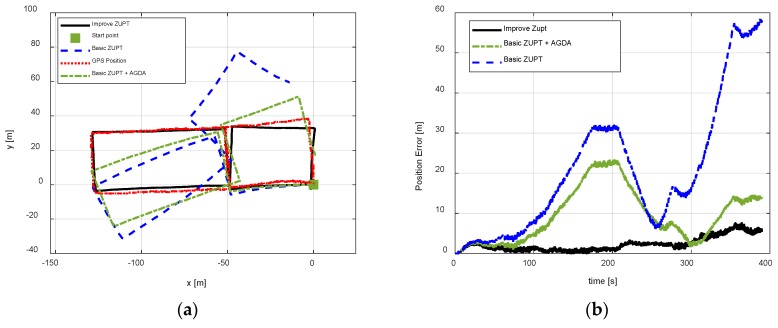
Position result for test 1. (**a**) Trajectory tracked result; (**b**) position error by different algorithms.

The first pedestrian trajectory conducted for testing the proposed algorithm is an “8 shape” path. Pedestrian remained standing for 7 s to attain initial alignment attitude at the beginning of the test. Figure 7a is the estimation result of attitude obtained by initial alignment. Figure 7b is the magnetic disturbance estimation result during the initial alignment period. Figure 8 and Figure 9 are the heading estimation results, and the pedestrian positioning result based on the basic ZUPT and the improved ZUPT respectively. The standard position of GPS is shown in Figure 9a. Figure 9b is the position error, which is the difference between the position calculated by MIMU position and GPS position. In addition, the legends meaning of the curves in figures are summarized as follows:

- Basic ZUPT: navigation algorithm of the basic ZUPT algorithm is discussed in Section 2.2;
- Fast-initial alignment by AGDA: ADGA initial alignment algorithm proposed in Section 3;
- Basic ZUPT + AGDA: the ZUPT algorithm based on the AGDA fast-initial alignment algorithm in Section 2.2 and the navigation algorithm by basic ZUPT in Section 2.2;
- Improve ZUPT: the ZUPT algorithm based on the fast-initial alignment algorithm by AGDA was introduced in Section 2.2 and the navigation algorithm by the adaptive positioning algorithm discussed in Section 4;
- GPS Position: GPS position as the standard reference information.

The curves of left column in Figure 7a are the attitude result during the alignment period from AGDA initial alignment algorithm, basic ZUPT algorithm and MTi-G710 output respectively. The curves of the right column in Figure 7a are the attitude error, which is the difference between the AGDA and reference attitude, the basic ZUPT and the reference algorithm. For the MTi-G710, the attitude can be obtained by the coupled navigation method when GPS available and the motion is not severe. Therefore, when the pedestrian stands on the ground for initial alignment, the attitude from MTi-G710 is used as the reference information to judge the initial alignment performance.

From the curves in Figure 7a it can be seen that, the attitude estimation results calculated by AGDA are −3.075°, 6.501°, −3.037°. The convergence time of initial alignment is less than 5 s which meets the requirement of fast alignment for FIPPS. In addition, the horizontal attitude calculated by the basic ZUPT and AGDA are the same as each other. Because the velocity error is the observation for Kalman filter for basic ZUPT, and the horizontal attitude are calculated by the component of gravity measured by accelerometers for AGDA. Therefore, the source information of calculating the two groups of horizontal attitudes are all acceleration from accelerometers. The heading accuracy calculated by AGDA is better than that by basic ZUPT because according to the conclusion in Section 3, the heading misalignment angle cannot observed and estimated by basic ZUPT, but it can be improved by AGDA, which takes the magnetometer as the reference sensor to estimate the heading attitude.

The output of the MTi-G710 magnetometer is in arbitrary units (a.u.), where one a.u. is the magnetic field strength during calibration at the Xsens calibration lab. Hence, there is no unit on the y-axis in Figure 7b. Mostly, the evaluation method is the position accuracy after magnetic disturbance compensated during the pedestrian walking stage (Figure 9).

From Figure 8, it can be concluded that the horizontal attitudes calculated by two different algorithms are nearly the same. The test results well fit with the theoretical conclusions in Section 2 and Section 4. The source information for calculating the horizontal attitudes are the acceleration sensed by accelerometers, whether basic ZUPT or AGDA is introduced as the correction method. The heading information in Figure 8c by two different algorithms is different. Because the heading misalignment angle obtained by improved ZUPT was estimated after the magnetic disturbance was compensated during the pedestrian walking stage. However, an accumulated heading error is produced by basic ZUPT because the heading misalignment angles cannot be estimated. Therefore, the problem of unobserved heading misalignment for FIPPS ZUPT is solved by introducing the Inertial/Magnetometer positioning algorithm based on an adaptive Kalman Filter, and the magnetic disturbance is compensated at the same time. Then, the position error can be reduced, which is shown in Figure 9.

The pedestrian position result is shown in Figure 9 for test 1. The figure compares the performance of three algorithms. ZUPT and AGDA was able to perform better than the basic ZUPT and positional errors are reduced. It was effective to introduce an initial alignment algorithm for FIPPS. The best pedestrian position test results were achieved by the proposed improve ZUPT as compared with the other two algorithms. The pedestrian trajectory by improve ZUPT can be tracked better using adaptive inertial/magnetometer positioning algorithm and introducing the heading misalignment angle as the observation, whereas, the pedestrian trajectory cannot be tracked by basic ZUPT, which is embodied in two aspects. One is increasing the initial heading error at the start point, and the other is accumulated heading error during the pedestrian walking period. One of the major reasons for the accumulated heading error is the unobserved heading misalignment angle during the pedestrian standing and walking period. Therefore, the performance of the pedestrian positioning is improved as it is based on the initial alignment algorithm by AGDA and inertial/magnetometer integrated positioning algorithm by compensating magnetic disturbance and the application of adaptive Kalman Filter.

Test 2

**Figure 10:**
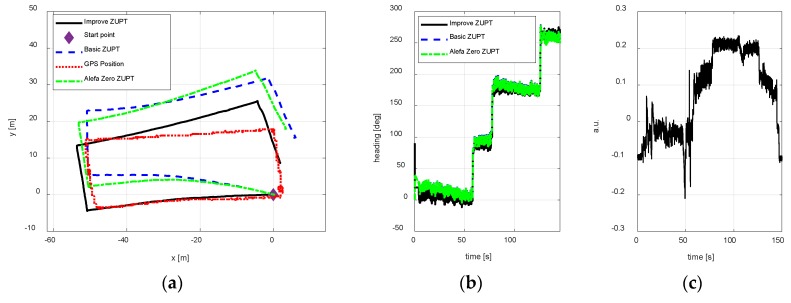
A rectangle path position result. (**a**) Trajectory; (**b**) Heading estimation result; (**c**) Magnetometer disturbances.

**Figure 11:**
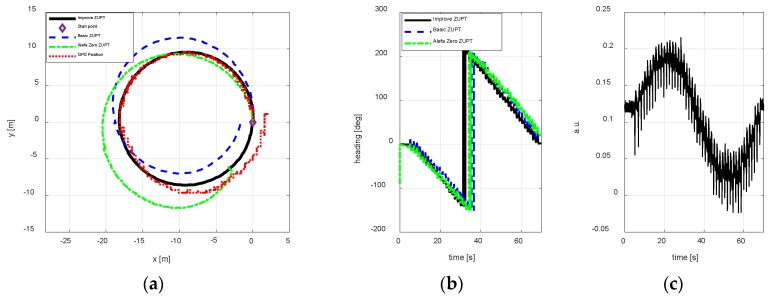
A circular path position result. (**a**) Trajectory; (**b**) Heading estimation result; (**c**) Magnetometer disturbances.

**Figure 12:**
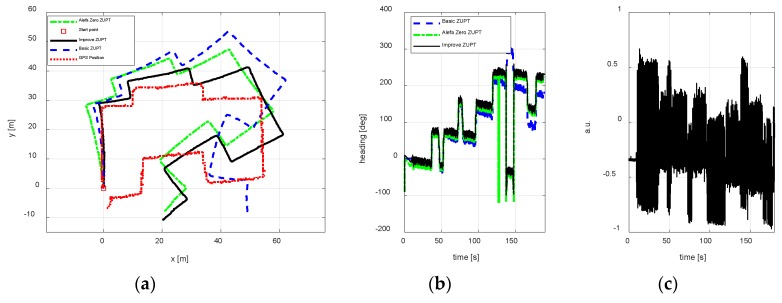
An irregular shape position result. (**a**) Trajectory; (**b**) Heading estimation result; (**c**) Magnetometer disturbances.

To test, whether the improve ZUPT algorithm proposed in this paper can be used in the changing magnetic interference environment, three test trajectories were carried out. The walking paths are rectangle, circle, and irregular shape respectively. Figure 10, Figure 11 and Figure 12 show the test results using different algorithms and GPS positions. The purpose of the proposed algorithm in this paper is reducing the position error of FIPPS by estimating and compensating the heading misalignment angles. The trajectory and the heading tracked result are shown in figures. The accuracy of GPS is 1m/s, and the sample is 1 s. The legends used in the figures are summarized as follows:

- Basic ZUPT: the basic ZUPT algorithm is discussed in Section 2.2;
- Alefa Zero: ZUPT algorithm based on the adaptive positioning algorithm is discussed in Section 4, but α=0 in Equation (9c). It means that the heading misalignment angle is introduced as the observation for Kalman Filter, but the magnetic disturbances were not compensated.
- Improve ZUPT: ZUPT based on the fast-initial alignment algorithm by AGDA in Section 2.2 and the navigation algorithm by the adaptive positioning algorithm in Section 4.
- GPS Position: GPS position as the standard reference information.

According to the magnetometer principle, it can be obtained that the sum of the magnetometer measurement vector along three axes is constant if there are no magnetic disturbances. The magnetic vector sum will change when a magnetic disturbance appears. In addition, the output of the MTi-G710 magnetometer is processed in arbitrary units (a.u.) in XSENS’s lab. Therefore, the difference between the vector sum of the magnetometer measurement along three axis and 1 is the magnetic disturbances during the experiments. The differences are shown in Figure 10c, Figure 11c and Figure 12c to illustrate the magnetic disturbance degree for the three tests.

The position error is reduced by estimating the heading misalignment angle and using the adaptive Kalman Filter proposed in this paper, hence, the trajectory tracked and the heading estimation results are the two main states to reflect the performance of the algorithm proposed in this paper. According to the test results of different walking paths, it can be observed the position accuracy of the proposed adaptive inertial/magnetometer positioning algorithm is better than basic ZUPT and Alefa Zero ZUPT algorithms, and the trajectory can be tracked better. However, the trajectory positional tracking result is worse as compared to the test 1 because the magnetic interference is changing in test 2, and only part of the magnetic noise can be compensated by the adaptive Kaman Filter. The unobserved magnetic noise leads to the accumulating heading error and the position error. 

## 6. Conclusions

An improved method for foot-mounted inertial/magnetometer pedestrian positioning based on an adaptive gradient descent algorithm is proposed in this paper. According to the principle of foot-mounted inertial pedestrian positioning, this paper presents a fast-initial alignment algorithm based on AGDA, where the initial attitude and the magnetic disturbance can be estimated during the pedestrian standing period by introducing the AGDA as the alignment algorithm. To solve the problem of the heading unobserved misalignment angle, this paper presents an adaptive inertial/magnetometer positioning algorithm. The magnetic disturbance is compensated, and the heading misalignment angle is used as an observation, therefore the accuracy of pedestrian position is improved. In addition, to compensate the change in the magnetic disturbance for ZUPT an adaptive parameter in the Kalman filter is added to enhance the positioning solution. MTi-G710, which is produced by XSENS is used to verify the proposed technique. The experimental results positively demonstrate the effectiveness of the proposed method for pedestrian-positioning in this paper.

## Figures and Tables

**Table 1:** MTi-G710 Parameters.

Sensors			Typical	Max
Gyro	Bias repeatability	[deg/s]	0.2	0.5
	Noise density	[deg/s]	0.01	0.015
Accelerometer	Bias repeatability	[m/s^2^]	0.03	0.05
	Noise density	[μg/√Hz]	80	150

## Appendix A

A quaternion is a four-dimensional complex number that can be used to represent the orientation of a ridged body or coordinate frame in three-dimensional space. The relationship between the $q={\left[\begin{array}{cccc} q_{1} & q_{2} & q_{3} & q_{4} \end{array}\right]}^{T}$ and the corresponding rotation matrix $C_{n}^{b}$ can be obtained:

(A1) $$C_{n}^{b}=\left[\begin{array}{ccc} 2q_{0}^{2}+2q_{1}^{2}-1 & 2q_{1}q_{2}+2q_{0}q_{3} & 2q_{1}q_{3}-2q_{0}q_{2}\\ 2q_{1}q_{2}-2q_{0}q_{3} & 2q_{0}^{2}+2q_{2}^{2}-1 & 2q_{2}q_{3}+2q_{0}q_{1}\\ 2q_{1}q_{3}+2q_{0}q_{2} & 2q_{2}q_{3}-2q_{0}q_{1} & 2q_{0}^{2}+2q_{3}^{2}-1 \end{array}\right]$$

Let $\left[\begin{array}{ccc} {\tilde{\varphi }}_{x} & {\tilde{\varphi }}_{y} & {\tilde{\varphi }}_{z} \end{array}\right]$ be Euler angles usually defined in inertial navigation applications: the first rotation is about the z-axis through an angle ${\tilde{\varphi }}_{z}$ (heading angle), the second is about the y-axis through an angle ${\tilde{\varphi }}_{y}$, and the third is about the x-axis through an angle ${\tilde{\varphi }}_{x}$ from n frame to b frame. When the Euler angles are $\left[\begin{array}{ccc} {\tilde{\varphi }}_{x} & {\tilde{\varphi }}_{y} & {\tilde{\varphi }}_{z} \end{array}\right]$, the corresponding rotation matrix $C_{n}^{b}$ can be obtained:

(A2) $$\begin{array}{l} C_{n}^{b}=C_{x}C_{y}C_{z}\\ =\left[\begin{array}{ccc} 1 & 0 & 0\\ 0 & \cos{\tilde{\varphi }}_{x} & \sin{\tilde{\varphi }}_{x}\\ 0 & -\sin{\tilde{\varphi }}_{x} & \cos{\tilde{\varphi }}_{x} \end{array}\right]\left[\begin{array}{ccc} \cos{\tilde{\varphi }}_{y} & 0 & -\sin{\tilde{\varphi }}_{y}\\ 0 & 1 & 0\\ \sin{\tilde{\varphi }}_{y} & 0 & \cos{\tilde{\varphi }}_{y} \end{array}\right]\left[\begin{array}{ccc} \cos{\tilde{\varphi }}_{z} & \sin{\tilde{\varphi }}_{z} & 0\\ -\sin{\tilde{\varphi }}_{z} & \cos{\tilde{\varphi }}_{z} & 0\\ 0 & 0 & 1 \end{array}\right]\\ =\left[\begin{array}{ccc} \cos{\tilde{\varphi }}_{y}\cos{\tilde{\varphi }}_{z} & \cos{\tilde{\varphi }}_{y}\sin{\tilde{\varphi }}_{z} & -\sin{\tilde{\varphi }}_{y}\\ \sin{\tilde{\varphi }}_{x}\sin{\tilde{\varphi }}_{y}\cos{\tilde{\varphi }}_{z}-\cos{\tilde{\varphi }}_{x}\sin{\tilde{\varphi }}_{z} & \sin{\tilde{\varphi }}_{x}\sin{\tilde{\varphi }}_{y}\sin{\tilde{\varphi }}_{z}+\cos{\tilde{\varphi }}_{x}\cos{\tilde{\varphi }}_{z} & \sin{\tilde{\varphi }}_{x}\cos{\tilde{\varphi }}_{y}\\ \cos{\tilde{\varphi }}_{x}\sin{\tilde{\varphi }}_{y}\cos{\tilde{\varphi }}_{z}+\sin{\tilde{\varphi }}_{x}\sin{\tilde{\varphi }}_{z} & \cos{\tilde{\varphi }}_{x}\sin{\tilde{\varphi }}_{y}\sin{\tilde{\varphi }}_{z}-\sin{\tilde{\varphi }}_{x}\cos{\tilde{\varphi }}_{z} & \cos{\tilde{\varphi }}_{x}\cos{\tilde{\varphi }}_{y} \end{array}\right] \end{array}$$

## Appendix B

Adjusting Equation (7), an expression of $\cos\varphi_{0}$ and $\sin\varphi_{0}$ are obtained as follows:

(A3) $$\{\begin{array}{l} \cos\varphi_{0}=\frac{m_{x}^{\tilde{n}}-H\cos{\tilde{\varphi }}_{z}}{\delta m_{xy}}\\ \sin\varphi_{0}=\frac{m_{y}^{\tilde{n}}-H\sin{\tilde{\varphi }}_{z}}{\delta m_{xy}} \end{array}$$

Squaring two sides of Equation (A3) and making the sum, the following expression is obtained:

(A4) $$m_{x}^{\tilde{n}}\cos{\tilde{\varphi }}_{z}+m_{y}^{\tilde{n}}\sin{\tilde{\varphi }}_{z}=\frac{m_{x}^{\tilde{n}}{}^{2}+m_{y}^{\tilde{n}}{}^{2}+H^{2}-\delta m_{xy}^{2}}{2H}$$

Put $\sin\chi =\frac{m_{x}^{\tilde{n}}}{\sqrt{m_{x}^{\tilde{n}}{}^{2}+m_{y}^{\tilde{n}}{}^{2}}}$ and $\cos\chi =\frac{m_{y}^{\tilde{n}}}{\sqrt{m_{x}^{\tilde{n}}{}^{2}+m_{y}^{\tilde{n}}{}^{2}}}$, and taking them into Equation (A4), the following equation is obtained:

(A5) $$\sqrt{m_{x}^{\tilde{n}}{}^{2}+m_{y}^{\tilde{n}}{}^{2}}\left(\frac{m_{x}^{\tilde{n}}}{\sqrt{m_{x}^{\tilde{n}}{}^{2}+m_{y}^{\tilde{n}}{}^{2}}}\cos{\tilde{\varphi }}_{z}+\frac{m_{y}^{\tilde{n}}}{\sqrt{m_{x}^{\tilde{n}}{}^{2}+m_{y}^{\tilde{n}}{}^{2}}}\sin{\tilde{\varphi }}_{z}\right)=\frac{m_{x}^{\tilde{n}}{}^{2}+m_{y}^{\tilde{n}}{}^{2}+H^{2}-\delta m_{xy}^{2}}{2H}$$

Using the trigonometric function equation to Equation (A5):

(A6) $$\sin\left(\chi +{\tilde{\varphi }}_{z}\right)=\frac{m_{x}^{\tilde{n}}{}^{2}+m_{y}^{\tilde{n}}{}^{2}+H^{2}-\delta m_{xy}^{2}}{2H\sqrt{m_{x}^{\tilde{n}}{}^{2}+m_{y}^{\tilde{n}}{}^{2}}}$$

The calculation formula of ${\tilde{\varphi }}_{z}$ in Equation (8) is obtained by adjusting Equation (A6). Then, the calculation formula of $\varphi_{0}$ in Equation (8) is obtained with the same method.
